# The Differential Impact of the COVID-19 Crisis on Small-Scale Development Initiatives, a Cross-Country Comparison

**DOI:** 10.1007/s11266-021-00385-z

**Published:** 2021-09-07

**Authors:** Sara Kinsbergen, Marieke Pijnenburg, Tom Merlevede, Luca Naus, Dirk-Jan Koch

**Affiliations:** Anthropology and Development Studies, Radboud University, Nijmegen, The Netherlands

**Keywords:** COVID-19 crisis, Private development initiatives, Belgium, Denmark, France, The Netherlands, Cross-country comparison

## Abstract

The COVID-19 pandemic presents Northern-based development organisations with unprecedented difficulties. They are challenged in fundraising opportunities in their home countries and in finding ways to continue their work in the Global South. As the first study to present a systematic mixed method, cross-country study of small-scale, voluntary development organisations in four different European countries, this study provides insight into the role of these private development initiatives (PDIs) in the COVID-19 crisis and sheds light on the differential impact of the crisis on these organisations. Whereas most PDIs are involved in long(er)-term development interventions, the COVID-19 crisis was for most organisations their first experience of emergency aid. Overall, we see strong resilience among PDIs and also find that the organisations which relied more exclusively on traditional methods of fundraising (offline) received a greater funding hit than organisations—often with more younger members—that had already moved to online fundraising.

## Introduction

Since the moment the COVID-19 pandemic started to affect countries in the Global North in March 2020, a wide variety of studies started to examine its impact on various sectors. While civil society organisations where busy arranging their COVID-19 response, either targeting people in their home countries or in the Global South, several studies started analysing the impact of the crisis on the organisations themselves.

In the UK for example, 94% of the charitable organisations mentioned a negative impact on their financial situation following the crisis, especially resulting from a significant decrease of public donations, fundraising and earned income. Following this, in September 2020, over a quarter of UK organisations had made staff redundant, and a fifth reported that they expected to have to make redundancies when the government Job Retention Scheme ended by the end of October. A large majority of organisations expects the crisis to have a negative impact on their ability to deliver on their objectives (Pro Bono Economics, 2020).

In the Netherlands, 62% of charitable organisations experienced an income decrease between January and September 2020. On average, organisations experienced an income decrease of 6% of their annual income, and over half of the organisations expected this negative trend to continue between September and December 2020. As in the UK, the income loss largely resulted from a decline in private donations. Unlike the UK, only 10% of the organisations had made staff redundant. A majority of the organisations started COVID-19 activities (Schulpen et al., 2020). Overall, it is being concluded that hitherto charitable organisations, despite experiencing serious challenges, are showing great resilience.

Studies so far also show how the crisis has a differential impact on different types of charitable organisations (Schulpen et al., 2020; PBE, 2020; SIDCN, 2020). First of all, the charitable cause determines how organisations are being impacted by the COVID-19 crisis, with in the Netherlands health-related organisations facing a significant harder financial drawback and nature and environmental organisations experiencing an overall increase in budget. Also, organizational characteristics turn out to define the impact of the crisis. Both in the Netherlands and in the UK, smaller organisations are, overall, more severely hit by the crisis, and a larger number of them do not expect to survive the crisis (Schulpen et al., 2020; PBE, 2020; SIDCN, 2020). In the Netherlands, these organisations are also more negative about the ability to continue their regular work in the near future and, compared to larger organisations, a smaller number of them started COVID-19 activities (Schulpen et al., 2020). In the study at hand, we zoom in on small civil society organisations—with civil society referring to the ‘third sector’ of society, distinct from government and business and including the family and the private sphere. In particular, we focus on small-scale, voluntary development organisations (henceforth, PDIs). We study the impact of the COVID-19 crisis on these so-called private development initiatives across four European countries: Belgium, the Netherlands, Denmark and France and study the role they play in the Global South in response to the crisis.

For decades, development efforts in the Global South have been considered—by policymakers as well as academic researchers—as the exclusive domain of intergovernmental organisations (such as the UN) and non-governmental organisations (NGOs) in the Global North (Schulpen & Huyse, 2017). Yet, in the past decade, the international development sphere has undergone significant changes, including an increase in the number and diversity of actors (Richey & Ponte, 2014). Over the years, it has become quite clear that the community is becoming increasingly fragmented (Develtere, 2012).

One specific set of alternative development actors that entered the development community are private development initiatives, which are also commonly referred to as citizen initiatives. Generally, these organisations are defined by their small-scale and voluntary character. As noted by Appe and Tech (2019), these PDIs are ‘an expanding subset of international NGOs working in private development aid which are based in the Global North and fund foreign aid projects in the Global South’ (p. 2). With exception from academic research in the Netherlands, studies on PDIs do not employ a clear-cut definition of what defines a PDI. In the Netherlands, ‘small-scale’ is defined as having fewer than 20 regular staff members, or an annual budget of less than 1 million euros, and their voluntary character is of an upper limit of 20% or less of paid staff members (Kinsbergen & Schulpen, 2009, p. 166).^1^

A European mapping in 17 countries concluded that, although differently named and in different numbers, citizen initiatives are common and widespread across Europe (Pollet et al., 2014). Outside Europe, in the USA and Canada, there is also an increasing number of PDIs, but the current study will focus on PDIs in European countries (Appe & Schnable, 2019; Davis, 2020).

Whereas PDIs are commonly known for their role as development actors contributing to long(er)-term development processes (Haaland and Wallevik 2019), this current study analyses the role of PDIs in a humanitarian crisis. More precisely, it questions whether and how PDIs responded to the COVID-19 crisis in the countries where they operate and how they were impacted by this crisis. We contribute to the current understanding of the impact of COVID-19 on charitable organisations by focusing on this particular group of organisations and by answering the following question: which factors explain why certain organisations are hit harder than others and, following this, how can organisations mitigate the impact of the crisis? Before presenting these findings, a sketch of the PDI landscape in the different countries of study will be presented. After the findings—in the discussion and conclusion section—the validity of the findings will be debated and policy implications proposed.

## Methodology

This study was conducted in close cooperation with a European network of civil society organisations that provide financial and/or non-financial support to PDIs. Currently, the network consists of support organisations in Belgium (4de Pijlersteunpunt, eu can aid! and the Province of West-Flanders), Denmark (CISU), France (La Guilde) and the Netherlands (Stichting Wilde Ganzen, Vastenactie and Partin). Nine out of eight organisations of the network participated in the study.^2^ The selection of countries of study was thus the result of the network composition. All of these organisations provide different types of support to the PDIs in their respective countries and/or in the Global South.

The study took up a mixed method design. Data collection took place between June and November 2020. To gain insight into the context wherein PDIs operate in the different countries in the Global North, 15 in-depth interviews took place with core staff members (occupying functions such as ‘responsible south operation’, ‘communication consultant’ and ‘head of the PDIs unit’) of the eight different support organisations. In addition, previous studies were analysed. The interviews and desk study allowed to gain understanding of the field of PDIs in the different countries and the support provided to PDIs during the COVID-19 pandemic by the support organisations. These insights gave as well guidance to the design of the survey (see ‘Appendix 1’). Insights into the response of PDIs to the COVID-19 crisis were gained through project administration files provided by the four PDI support organisations that provided financial support during the COVID-19 crisis (La Guilde, Province of West-Flanders, CISU and Stichting Wilde Ganzen).

To understand the impact of the crisis on PDIs and how they responded, a survey was conducted among PDIs in the participating countries (see ‘Appendix 1’ for the survey). The survey was distributed via the support organisations through email, newsletters and social media to a total of 4930 organisations. In total, 567 surveys were completed, resulting in a response rate of 11.5%. Dependent on a variety of factors, an average response rate of around 33% can be expected for online surveys with a standard deviation of 22% (Nulty, 2008; Shih & Fan, 2009). Table 1 presents the response rate for the different countries. Because the Danish support organisation CISU also supports larger development organisations and does not use a strict definition of PDIs, in agreement with CISU we applied the Dutch PDI definition, meaning an upper annual budget limit of 1 million euro, and 15 organisations were thus excluded from the study.

**Table 1:** Survey sampling

Country	Reach	*N*	Response rate (%)
Belgium	1047	113	10.8
Denmark	268	84	31.3
France	1000	106	10.6
The Netherlands*	2615	264	10.1

*The network of Wilde Ganzen, Vastenactie and Partin partly overlaps. This most probably results in a biased response rate for the Netherlands

We used the survey data to understand whether and how country differences and organisational characteristics are of influence on the COVID-19 response of PDIs and the impact of the crisis on these organisations. Preceding this, we introduce our sample and highlight some of the important differences between PDIs in the different countries. In this part, we include all of the organisations that participated in the survey (*n* = 567). Following this, we explore if organisational characteristics, such as age of the organisation, number of members, percentage of paid staff, annual budget, revenue sources and fundraising strategies to be of influence. We start the analysis with multiple bivariate analyses for three dependent variables: impact of the crisis on organisations, experienced concerns and opportunities. Next, we zoomed in on the impact of the crisis on PDIs’ budget. Here we only included those PDIs that provided information on the relevant variables, resulting in 428 organisations being included in this part of the analysis. Considering we treated our dependent variable, impact on budget, as a continuous variable and the distribution of the variable, we applied a standard multiple regression analysis.

Finally, both to enlarge our understanding of PDIs’ role in the crisis and the way they were impacted by it, in-depth, semi-structured interviews with core members of 12 PDIs in Belgium (4) and the Netherlands (8) took place. For more information on the organisations that participated in this part of the study, see ‘Appendix 2’. All interviews were conducted in October 2020, took approximately 1–2.5 h and were held online. The interviews comprised three parts: (1) a general introduction to the organisation, (2) the impact of COVID-19 on the organisation, (3) the response of the organisation to the crisis. The data obtained by these interviews were analysed by using an open coding strategy. To prevent further complications to the participation of PDIs in this part of the study, we decided to conduct interviews with only Dutch/Flemish-speaking PDI members (the mother tongue of the researchers), limiting this part of the study to PDIs in Belgium and the Netherlands. The organisations were selected from the survey respondents. When sampling the organisations, we aimed for a group of PDIs reflecting the variety both in terms of organisation characteristics, type of interventions and response to and impact of the crisis (see ‘Appendix 2’ for more elaborate information on the sample).

## PDIs at a Glance

Based on the findings of the survey, we can conclude that, in all countries, citizen-led development organisations can overall be defined as (relatively) small-scale, voluntary organisations in terms of annual budget and number of (voluntary) members (see Table 2 for an overview of the main features of PDIs in the different countries). With an average number of 35 members and an annual budget of around EUR 147,000, Danish PDIs are by far the largest in terms of budget and number of core members compared to those in the other countries. With Belgium and France having 13 and 17 members on average, Dutch PDIs are the smallest with an average of 10 members per PDI. Dutch PDIs furthermore on average also have the smallest annual budget (EUR 51,331), followed by French (EUR 56,599) and Belgian PDIs (EUR 63,503).

**Table 2:** Main features of PDIs in the different countries

	The Netherlands	Belgium	Denmark	France
	Particuliere initiatieven *(Private Development Initiatives)*	Flanders: 4de pijler-organisaties *(4th Pillar-organisations)* Wallonia: Initiatives Populaires de Solidarité Internationale	No specific terminology	Association de Solidarité Internationale *(International solidarity association)*
	10 members	13 members	35 members	17 members
	Volunteers: 92.4% Paid staff: 7.6%	Volunteers: 96.6% Paid staff: 3.4%	Volunteers: 90.6% Paid staff: 9.4%	Volunteers: 92.9% Paid staff: 7%
	Annual budget: €51,331,-	Annual budget: €63,503,-	Annual budget: €147,123,-	Annual budget: €56,599,-
	± 5000 PDIs	1500–6400 PDIs	Unknown	4000 PDIs
	1. Private individuals 2. NGOs 3. Private foundations	1. Private individuals 2. Government grants 3. Schools	1. NGOs 2. Private individuals 3. Private foundations	1. NGOs 2. Private individuals 3. Government grants
	1. Donations via website 2. Direct mailings 3. Social media campaigns	1. Organising events 2. Direct mailings 3. Direct sale of products	1. Donations via website 2. Organising events 3. Direct mailing	1. Organising events 2. Collections 3. Direct mailings
	1. Uganda 2. Ghana 3. Indonesia	1. DRC (Congo) 2. Kenya 3. India	1. Kenya 2. Uganda 3 Tanzania	1. Burkina Faso 2. Senegal 3. Benin

Danish PDIs are also more professionalised, indicated by their relatively high share of paid staff members (9.4%). Although PDIs in all countries share a voluntary character, this feature is most prominent among Belgian PDIs, with nearly 97% of the organisations being completely run by volunteers. Dutch and French PDIs can be placed in the middle, with on average 92.4% and 92.9% volunteers, respectively. We did not study the characteristics of PDI members in this current study, but previous studies have shown that whereas PDI members in Belgium, the Netherlands and France are on average of middle age, Danish PDIs tend to be more diverse in terms of age, with more younger people being involved as well (Kinsbergen et al., 2020a, 2020b, 2020c, 2020d).

With a total of 7573 volunteers, 526 paid staff and a total annual budget of 29,900,81 euro, the 541 European PDIs that participated in this study are active in 104 different countries in the Global South and are currently supporting a total of 885 development interventions. The four countries participating in this study all experienced a peak of new PDIs being established between 2007 and 2010. Compared to PDIs in the other countries, Danish PDIs are the oldest (average of 22.37 years), while those in France are the youngest (13.67 years), followed by the Belgian (14.34 years) and the Dutch (16.11 years) PDIs.

Although European PDIs can be found across the world, there is a strong concentration of PDIs operating on the African continent (71%) followed by nearly 30% being active in Asia and 8% in South America. Kenya (13.5%), Uganda (12%) and India (11.5%) are the three most important destination countries where PDIs operate. Danish PDIs seem to cluster mostly in East Africa, with Kenya, Uganda and Tanzania as their top project countries. French PDIs cluster mostly in West Africa, more specifically in Burkina Faso, Senegal and Benin. Dutch PDIs are most present in Uganda, followed by Ghana and Indonesia, and Belgian PDIs in the DRC, Kenya and India.

With a total of 259 PDIs active in 86 different countries, Dutch PDIs are the most omnipresent around the world. The 66 Danish PDIs in this study are spread over 50 different countries. The 103 French PDIs are active in 46 different countries and the 113 Belgian PDIs in 44 different countries.

**Fig. 1:**
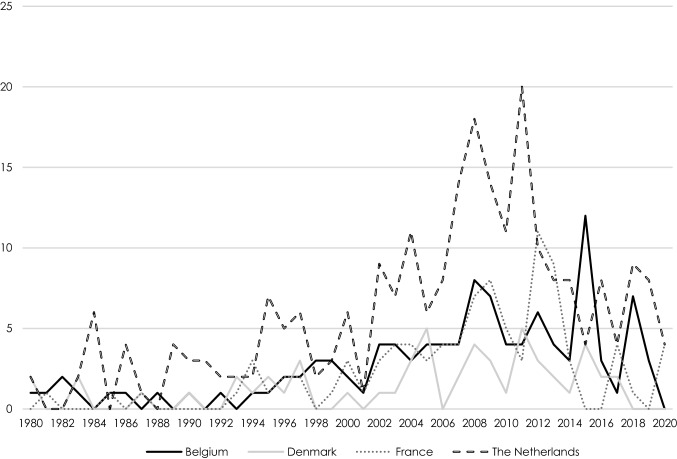
Establishment of PDIs per year

Figure 1 shows the importance of different revenue sources for PDIs. The overall picture shows that PDIs rely both on private donors and public, more institutionalised types of support. Belgian and Dutch PDIs depend mainly on private individuals, whereas PDIs in Denmark and France are most reliant on support provided by (established) NGOs, followed by donations by private individuals. For Dutch PDIs, NGOs are the second largest donors. Furthermore, both in Denmark and in the Netherlands, private foundations contribute to a considerable degree to the work of PDIs. In Belgium, government funding for PDIs stands out, with in particular municipalities and provinces are known to be highly supportive. Belgium also stands out with the strong support provided by schools.

PDIs undertake a wide variety of fundraising activities. First, it is apparent that PDIs in France, with 54.6%, depend to a large extent on direct (offline) fundraising activities. Dutch and Danish PDIs rely more on online fundraising activities, with, respectively, 61.3% and 61.8% of fundraising activities taking place online. In Belgium online and offline fundraising activities are almost equally important.

**Fig. 2:**
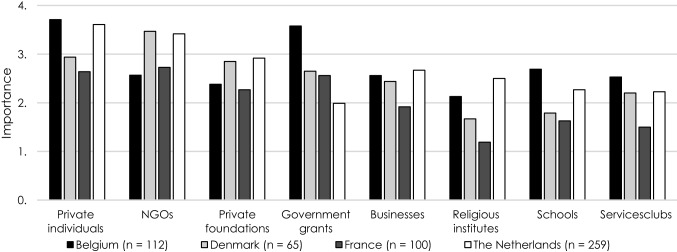
Most important revenue sources

Figure 2 provides an overview of the use of different fundraising activities by PDIs. It is striking that 83% of Belgian PDIs indicate they organise fundraising events, compared to only 44% of Danish PDIs. Belgian PDIs furthermore rely most on direct mailing and the sale of products. PDIs in the Netherlands tend to focus more on donations via their website and direct mailings for their funds, while French PDIs focus most on organising events and collections.

In response to the increasing number of PDIs over the years, the established development NGOs and governments in the different countries of study responded by implementing support systems for these organisations. The type and size of support provided differs across the countries of study and so does its organisational structure. It goes beyond the scope of this current study to describe and compare the support systems for PDIs in these countries in great detail; for a more in-depth analysis of this topic, see Pollet (2017) and Kinsbergen et al. (2020a, 2020b, 2020c, 2020d). What is of importance here is the more or less shared rationale underlying the policy resulting in the support systems. Established development actors in the Global North recognise the (potential) value PDIs can take up in their own communities (Kinsbergen & Molthof, 2021). PDIs are considered an expression of public support for development cooperation, and at the same time, ‘in many cases, governmental policies consider citizen initiatives as vehicles of public support for international development’ (Pollet, 2017, p. 189). It is expected that, because PDIs are strongly embedded with local communities in the Global North, they are able to reach out those parts of society that are not (yet) convinced of the importance of international development cooperation. As a result, most subsidy schemes request that PDIs organise public gatherings and raise attention about their work in local media in return for the project funding they received (Pollet, 2017, p. 189).

## The Impact of COVID-19 on PDIs

In this section, we first introduce the quantitative data, starting with a description of the (financial) impact of the crisis on PDIs and a reflection on the experienced concerns and opportunities related to the crisis. Thereafter we analyse what determines the impact of the crisis on PDIs by looking at the core characteristics of organisations and country differences. Subsequently, we present the findings of the interviews with PDI members.

The upper part of Table 3 presents the descriptive statistics of the main features of our sample, and the lower part shows the descriptive data on the impact of the crisis and the experienced concerns and opportunities.

**Table 3:** Descriptive statistics (*N* = 428)

	*M*	%	SD
**Organisational characteristics**			
Core characteristics			
Age (0–115yrs)	15.85		12.86
Annual budget 2019 (€ 0–739,247)	66,315.91		99,893.55
Volunteers (0–500)	13.21		28.50
Paid staff (0–54)	1.03		4.48
Amount of direct fundraising activities (0–4)	1.58		1.23
Amount of online fundraising activities (0–5)	2.02		1.50
Financial sources			
Private individuals			
Not important		21.26	
Important		78.74	
Businesses			
Not important		61.68	
Important		38.32	
Schools			
Not important		72.20	
Important		27.80	
Government grants			
Not important		59.11	
Important		40.89	
NGOs			
Not important		39.49	
Important		60.51	
Impact, opportunities & concerns			
Opportunities (1–5)	2.82		1.01
Concerns (1–4)	2.27		0.71
Impact crisis on budget (1–5)	2.18		1.13
Substantially decreased		35.51	
Somewhat decreased		27.57	
Did not change		24.77	
Somewhat increased		7.94	
Substantially increased		4.21	
Started new COVID-19 projects			
No		46.28	
Yes		53.72	
Impact COVID-19 on regular projects			
Stopped projects		24.73	
Continued projects less intensively		47.61	
Postponed projects to the future		62.50	
Offered activities online		17.82	
Accelerated projects		5.59	
Southern partner gained ownership		12.77	

### What Happened to Their Development Interventions?

The COVID-19 crisis directly affected 88% (*N* = 376) of PDIs’ regular projects and programmes between January and September 2020. Of these PDIs, nearly 25% had to stop (part of) their regular activities permanently. Around half continued their regular activities less intensively (47.6%) and/or postponed their regular activities (62.5%). Belgian PDIs more frequently (38.5%) permanently stopped (part of) their regular activities, followed by Danish (30.4%) and French PDIs (26.3%). Only 16.5% of Dutch PDIs decided to stop their regular activities. A small number of PDIs started offering their activities online (17.8%), and some accelerated the implementation of their regular activities (5.6%). Danish PDIs appeared to be most flexible in continuing to offer their activities online (37%), while those in Belgium (19.2%), France (15.8%) and the Netherlands (13.1%) did so to a lesser extent.

In addition to country differences, we find as well that organisations with a higher budget, are better positioned to continue their regular programs (*X*^2^ (2) = 6.17, *p* < .05). Also, the flexibility to adapt programs to an online version is depending on the resources of organisations. We find a significant positive relation between both the number of paid staff members (*X*^2^ (2) = 4.89, *p* < .05) and the annual budget of organisations (*X*^2^ (2) = 7.16, *p* < .05) and the possibility of organisations offering regular activities online.

Reasons to adapt or stop the (implementation of) regular activities ranged from limiting the health risks for local employees and/or the target group (60.4%), (travel) restrictions from authorities in project countries (58.2%) or in PDIs’ home countries (48.7%), to limiting health risks for employees and volunteers in PDIs’ home country (31.6%). In the fourth place, PDIs mention decreased income (35.9%) as a reason to stop or adapt their interventions. In particular younger PDIs (*X*^2^ (2) = 6.77, *p* < .05) and PDIs in Belgium (*X*^2^ (2) = 25.17, *p* < .001) were more likely to adapt their interventions due to decreased income. We also found that differences could be (partly) explained by PDIs’ core characteristics: PDIs with a higher annual budget (*X*^2^ (2) = 6.85, *p* < .05) and/or paid staff (*X*^2^ (2) = 5.11, *p* < .05) more often changed their projects to limit the health risks for local employees and/or their target group. Travel restrictions in project countries also had a significant negative effect on the continuation of regular activities for organisations with a higher annual budget, *X*^2^ (2) = 9.15, *p* < .01. PDIs with more volunteers more often changed their projects to limit health risks for employees/volunteers in the PDI’s home country, *X*^2^ (2) = 15.27, *p* < .001.

Over half of PDIs (61%) experienced increased demand for support from their local counterparts. In particular, partners of Danish PDIs made an increased appeal (71.2%) for support. While PDIs are mostly involved in long(er)-term development processes, over half of the PDIs (*n* = 202) mentioned they started COVID-19-related emergency projects.

We found a significant, positive relation between the budget of PDIs and the start of COVID-19 emergency projects: PDIs with a larger annual budget were more likely to have started COVID-19 emergency projects, *X*^2^ (2) = 9.42, *p* < .01. To get insight in the type and scope of PDIs’ response, we analysed the project databases of PDI support organisations that provided financial support to COVID-19-related projects. In the period between March and November 2020, CISU, La Guilde, Province of West-Flanders and Stichting Wilde Ganzen supported 322 PDIs with a total of 327 projects spread over 58 different countries, worth a total amount of over 5,390,000 euro. Together these projects aimed to reach just over two million beneficiaries. Of these projects, 68.2% focused on health (e.g. supply of face masks and alcohol gel) combined with food security (e.g. distribution of food packages). The thematic focus of projects was different across countries. Projects of Dutch PDIs mostly focus on either food security (35%) or health (35%), but less so combine both themes within one project (3%). Belgian (41%) and French PDIs (32%) on the other hand often combine food and health within one project or focus on health alone (23% and 17%, respectively), rather than food (0%; 3%). French PDIs furthermore focussed with 35% most on care and welfare projects, followed by Belgian (23%) and Dutch PDIs (9%).

The geographical focus of the PDIs’ COVID-19 emergency aid projects was largely on Africa. Of the 327 emergency aid projects, 221 (68%) were executed in Africa, with Kenya (*n* = 25) and Uganda (*n* = 23) having the largest concentration of interventions, followed by Burkina Faso (*n* = 17) Senegal (*n* = 14), Cameroon (*n* = 13) and South Africa (*n* = 12). With 17 projects, India holds the most projects in Asia, and Peru (*n* = 14) stands out in Latin America.

### The Differential Organisational Impact

Although many PDIs responded rapidly to the COVID-19 pandemic in solidarity with their partner organisations and the communities wherein they work, PDIs themselves were also heavily affected. Of the 428 organisations that participated in our study, overall, 63.1% experienced a decrease in income, 24.8% indicated their budget so far was not affected by the crisis and the budget of 12.1% of PDIs even increased.

**Fig. 3:**
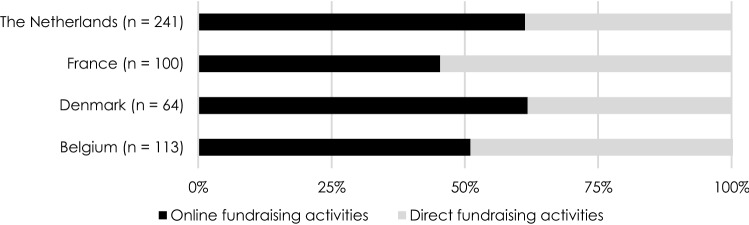
Online versus direct fundraising activities

The crisis impacted PDIs significantly differently in the countries of study, with medium to large effect size differences between the countries (*H*(3) = 38.96, Cohen’s *f* = .32)*.* While the majority of Danish PDIs did not experience a negative impact on their budget thus far, Belgian PDIs took the hardest hit with more than half of the PDIs’ budgets substantially decreased, followed by France and the Netherlands (see Fig. 3).

To understand what determines the impact of the crisis on PDIs budget, we conducted a regression analysis. Table 4 shows the results of the analyses. In order to be able to distinguish possible country differences, we included two different models. The first model tests the relation between the dependent variables we expected to be of influence and our dependent variable ‘impact budget’. The second model includes those same variables and contains the home countries of the PDIs. In this model, the Netherlands is included as the reference country.

**Table 4:** Regression analysis on impact budget (*N* = 428)

	Model 1		Model 2	
	B (SE)	Stand. B	B (SE)	Stand. B
(Constant)	2.443 (.179)			
Core characteristics				
Age	0.004 (.005)	0.048	0.001 (0.005)	0.012
Annual budget	4.37E−07 (0.000)	0.039	1.85E−07 (0.000)	0.016
Volunteers	− 0.002 (.002)	− 0.053	− 0.002 (.002)	− 0.046
Paid staff	− 0.003 (.012)	− 0.012	− 0.006 (.012)	− 0.025
Amount of direct fundraising activities	− 0.171*** (0.048)	− 0.187	− 0.138** (.049)	− 0.151
Amount of online fundraising activities	0.050 (.040)	0.066	0.036 (.041)	0.048
Financial sources				
Private individuals	− 0.160 (.14)	− 0.058	− 0.101 (.146)	− 0.037
Businesses	0.045 (.12)	0.019	0.019 (.119)	0.008
Schools	− 0.219 (.13)	− 0.087	− 0.144 (.132)	− 0.057
Government grants	− 0.230 (.112)	− 0.100	− 0.101 (.127)	− 0.044
NGOs	0.171 (.112)	0.074	0.022 (.121)	0.010
Countries (Netherlands = reference)				
Belgium			− 0.435* (.179)	− 0.158
Denmark			0.371 (.189)	0.108
France			− 0.152 (.167)	− 0.055
Adjusted *R*-square	0.049		0.072	

*< .05; **< .01; ***< .001

Interestingly enough, we do not find organisational characteristics, such as age or annual budget, to be of influence on the extent the crisis affected the budget of PDIs. Whereas we find (see above) that the number of resources (i.e. number of paid staff and budget) determines organisations’ ability to continue their regular programs, to adapt to online working and to start COVID-19 emergency projects, we do not find these factors to determine the financial resilience of PDIs. We do find that, compared to Dutch PDIs, Belgian PDIs are significantly harder hit by the crisis (*b* = − .435; *p* < .05). This might be resulting from a longer and stricter lockdown compared to the other countries having a more negative impact on the PDIs fundraising activities and (private) donors’ willingness to donate.

The results of the regression analyses show that while different revenue sources do not significantly determine the extent to which organisations’ budget is being affected, the type of fundraising strategies organisations undertake does determine the impact. We find a significant negative impact from direct fundraising activities (*b* = − .138; *p* < .01). Especially in Belgium and France, PDIs strongly rely on direct fundraising strategies with particularly Belgian PDIs organising a large number of fundraising activities in schools (see Fig. 1). Because of the lockdown, restricting or prohibiting public events and schools being closed during a longer period of time, a large number of fundraising activities were cancelled.

**Fig. 4:**
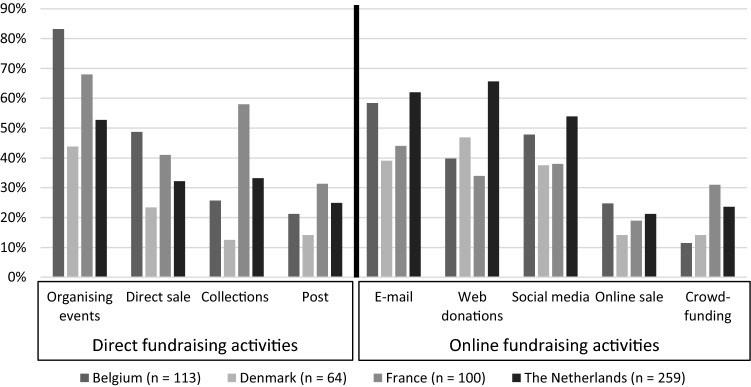
Importance of different fundraising activities

PDIs explained that their regular donors continued to support their work, and donors sometimes even increased their donations. Overall, we find that PDIs especially experienced a severe loss in income from direct fundraising activities (see Fig. 4). PDIs did not manage to compensate for this loss via online fundraising activities. The older age of PDI members might have hampered the organisations in developing and implementing a successful online fundraising strategy. Indeed, online fundraising activities merely functioned as a damage reduction strategy, as PDIs struggled to reach the status quo of normal financial circumstances. Only Danish PDIs managed to generate increased revenues via online mailings and social media campaigns.

When asked how PDIs expected the crisis to continue to affect their income in the near future, 65.7% expected a decrease, one quarter expected the budget of the organisation to remain stable, and 8.3% anticipated an increase in income.

**Fig. 5:**
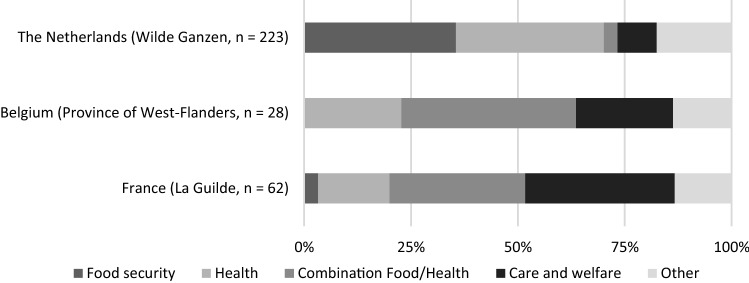
Thematic focus COVID-19 projects

Overall, we found that PDIs in France followed by those in Belgium were most concerned, while Danish and Dutch PDIs tended to be less concerned. With the majority of PDIs organising events, it comes as no surprise that PDIs are overall most concerned about the ability to organise fundraising activities. Belgian PDIs, especially, followed by French organisations, were most concerned about this perhaps due to their heavy reliance on direct fundraising activities. Danish PDIs expressed more concern related to upholding relationships with their local counterparts and meeting donor conditions. Interestingly enough, despite the negative (expected) impact of the crisis on PDIs’ financial situation and their interventions, overall, PDIs were not particularly worried about the continuation of their organisation or the survival of their local counterparts. French PDIs were more worried about this than those in other countries. Figure 5 shows an overview of the level of concern experienced by PDIs.

**Fig. 6:**
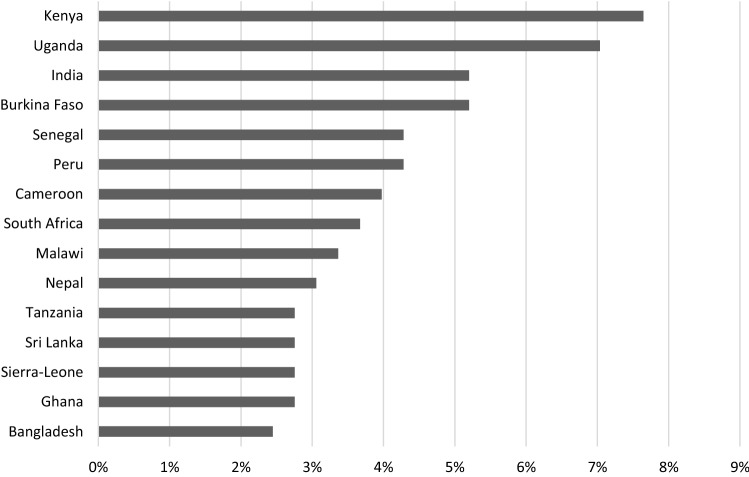
Top 15 project countries

Despite the negative impact of the crisis on PDIs, they also identified opportunities resulting from it. Overall, Danish PDIs saw the most opportunities resulting from the crisis, followed by Dutch PDIs. Most opportunities (see Fig. 6) were seen in increasing the local ownership of the partner organisation(s); 12.8% of PDIs indicated their partner had gained ownership to execute the project due to the COVID-19 crisis. Increasing local ownership has been a long-standing priority of PDIs but has been hard to implement in practice, and COVID might actually create a tipping point in this domain (Kinsbergen et al., 2020a, 2020b, 2020c, 2020d). There were no significant country differences here.

**Fig. 7:**
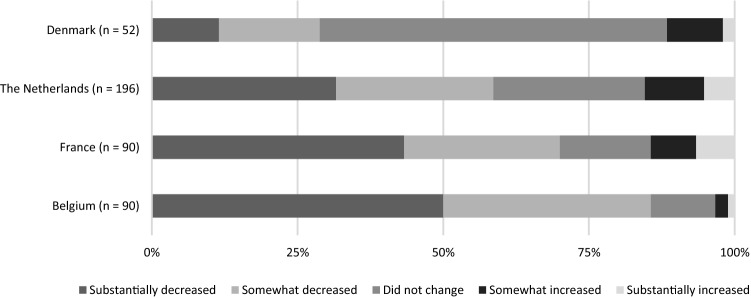
Impact of crisis on budget

**Fig. 8:**
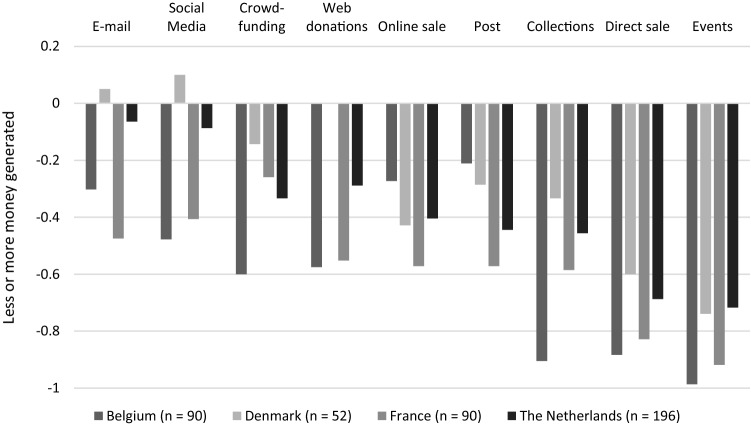
Change in income from fundraising activities

Although data do not allow to make firm statements on this, we expect that the severity of the COVID-19 crisis and the different measures taken by the governments in the different countries of study, might affect the impact of the crisis on PDIs and, related to this, the level of concern and opportunities expressed by PDIs. For example, compared to the Netherlands and Denmark, Belgium and France suffered a longer and more restrictive first lockdown in the spring of 2020 (e.g. restriction in travel distance, curfew, more limited number of visitors). Considering that these two latter countries strongly rely on physical fundraising events, this might explain the stronger negative impact of the crisis on the budget of PDIs in these countries. During the second and consecutive lockdowns in Europe, the policies were more in sync. Yet these episodes occurred after the end of the research (Figs. 7, 8).

### Impact Stories

The interviews with PDI members allow to deepen our understanding of both the response of PDIs to the crisis and the (expected) impact of the crisis on the organisations and their operations both in the global north and south.

Most of the PDIs describe how the COVID-19 regulations heavily affect their abilities to raise funds and therewith, their budget. One of the Belgian PDIs interviewed indicated that they had more than 20 festivals scheduled at the beginning of 2020 where they were planning to work in return for financial contributions to their PDI. Due to the COVID-19 crisis, all of these festivals were cancelled, and consequently, this organisation saw a big part of its income disappear (Interview PDI, case 11).

**Fig. 9:**
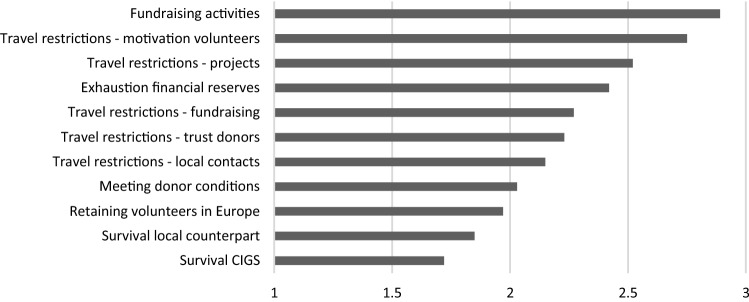
Level of concern. Values X-axis: 1. Not concerned, 2. Somewhat concerned, 3. Concerned

**Fig. 10:**
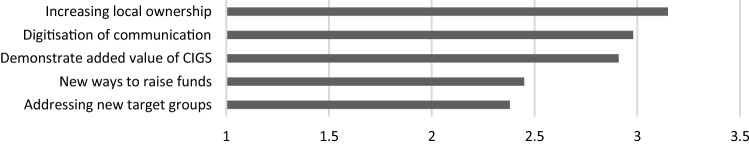
Level of opportunities. Values X-axis: 1. No opportunities–5. Many opportunities

Despite these financial challenges many of them experience, most interviewees describe how they immediately agreed to provide COVID-19 support to their partner organisations without even thinking about how to raise funds. When doing so, PDIs supported both interventions targeting the direct consequences of the COVID-19 crisis (e.g. distribution of hand sanitisers or masks) and projects responding to the consequences of the lockdown (e.g. food distribution). Their often-long-standing presence in specific regions and their durable partnerships with local organisations resulted in a strong commitment to respond (despite their own organisational challenges). It also allowed them to respond quickly. Their flexible funding base enabled them to make funds available in the short term or to generate new funding to finance the COVID-19 response of their counterparts (Figs. 9, 10).

While some of the regular projects PDIs were supporting before COVID-19 are currently running as before (e.g. construction work), other projects have experienced a more fundamental impact, as in the case of PDIs supporting schools or day-care centres. Where the school might have already reopened, parents are often no longer able to pay the tuition fees, preventing them from sending their children to school, but also affecting the financial self-reliance of the schools. All this results in some PDIs expressing feelings of insecurity. ‘It feels like all the efforts of the past years of both ourselves and our partner organisation have faded away’ (Interview PDI, case 11).

In addition to the impact of the crisis on the continuation of their regular projects and programs, some PDIs also expressed concern related to local ownership. They mentioned how the current crisis negatively affected the (financial) independence of their local counterparts and at times even stagnated or reversed exit strategies, as explained by the founder of one the PDIs:

Given our age, we had in mind to continue for a couple more years. We also discussed this during our last visit, that at a certain point, the ownership has to be handed over.… We had some ideas about this, but at the moment [due to the COVID-19 crisis], this is not at stake. (Interview PDI, case 8)

Others, however, describe how the enforced distance fastens the process of handing over responsibilities to local partners organisations.

PDIs do not only share concerns related to their work in the global south, and they also explain how travel restrictions to project countries negatively impact the motivation of PDI members. In the interviews, PDIs expressed that they would never think about quitting, but that they miss the positive energy resulting from visiting their local counterparts and projects. Not being able to see the results of their (fundraising) efforts makes the work more difficult:

I get the most energy when I am ‘in the field’. When I am there and I am busy with the kids, or making connections between people, that is my power. My passion is there in Brazil and I arrange the necessary stuff in the Netherlands. Thus, I did not get the positive energy that I always get from my annual visit and that is something that I missed. (Interview PDI case 3)

Next to this, PDI members also worry that a long-lasting COVID-19 crisis might impact the visibility of PDIs in their own communities. This does not only affect their fundraising activities, but also the role they play in contributing to global citizenship. During the interviews, one of the Belgian PDIs mentioned that because of this they also had fewer opportunities to inform people about the situation of people living in the Global South. That is, PDIs experienced the cancellation of these events as a threat to their role as contributors to global citizenship. This concern was also expressed by an interviewee:

In the beginning I had the impression the COVID-crisis had a positive effect on feelings of solidarity with our projects. After I sent the first mailing, people spontaneously donated money, but I don’t think that this will last. […] I also believe that these days people are more likely to take care of each other here [in Belgium]. The projects in the global South are far away for them, and we cannot organise events anymore to keep them involved. Normally, we would show up everywhere, on Christmas markets, everywhere (Interview PDI, case 11)

Especially for those countries where PDIs are subsidised because they are believed to contribute to strengthened public support for development cooperation and contributors to the notion of global citizenship, the crisis might result in a double burden: while impacting their work in the Global South, it also hampers them in reaching out to people in their own communities to involve them in the work of their organisations. Especially in Belgium and France, support organisations and PDIs themselves see a clear role for PDIs in their own society as contributors to global citizenship. This is being reflected in the fundraising strategies of such organisations in France and Belgium. This results in a stronger negative impact of the current crisis on the incomes of PDIs and prevents them from realising their goals in the Global South.

However, even though most PDIs indicate their organisations have been (heavily) influenced by the crisis, almost none of them question the survival of their organisation, and all of them are planning to continue their work in the future:

No way… There is really no way that we will let our partner organisation down now. They are getting back on track after the lockdown, and with all the problems going on now (Interview PDI, case 11)

## Conclusion and Discussion

This study provides insight into the role of citizen-led, small-scale, voluntary development organisations in the COVID-19 crisis and sheds light on the impact of the crisis on these organisations. The study took place among PDIs and their support organisations in Belgium (with a focus on Flanders), Denmark, France and the Netherlands. This study is the first to present a systematic cross-country study of PDIs in four different European countries. We found that the organisations are in general resilient, but the crisis had a differential impact, depending mostly on the relative size of the organisation and their fundraising strategies.

While especially known for their involvement in longer-term development interventions, this study shows that during a crisis PDIs are also playing their role. The findings show that, despite being impacted by the crisis, PDIs overall showed strong resilience, with many finding ways to continue their regular work and/or start COVID-19 interventions. PDIs have been able to mobilise significant resources to support their local counterparts in providing COVID-19 emergency aid. They remain dedicated to providing continued support to their local counterparts and the communities where they work. Whereas some PDIs paused their regular activities, most are confident they will be able to continue supporting their partners and their work in the near future. In this discussion, we first focus on the validity of those findings (both internal and external), followed by two policy implications. We conclude with some suggestions for future research.

With respect to external validity, one question that needs to be asked is how representative the PDIs of the four countries of study are compared to those from other countries, both in and outside of Europe. This question is especially relevant because our findings indicate that context matters. PDIs across countries share some common characteristics, but there are country differences; for example, Danish PDIs tend to be larger (in terms of budget and number of members), more professionalised (in terms of number of paid members) and older. These are characteristics that affect the impact of the COVID-19 crisis on the organisation and the role it takes up in the crisis.

The countries of study might also include a bias, because these countries might be considered ‘PDI champion countries’ with support systems for already in place. A mapping of 17 European countries pointed out the differences in terms of maturity of the PDI (support) sector, with Belgium, France and the Netherlands being more established than other European and non-European countries (Pollet, 2017). Appe and Schnable (2019, p. 1841) sought to partially explain the differences in weaknesses between US and Dutch PDIs in capacity-building programmes: ‘Unlike in the Netherlands, volunteer-run development organisations in the US have not been the target of financial support or capacity building from the national development agency or from large NGOs’. It might therefore be hard to generalise the findings of these four countries to other countries.

To determine the internal validity of the study, we scrutinised the representativeness of the sample of PDIs that participated, either via the survey or via the project databases of the support organisations. Because we approached PDIs via support organisations, it could be that younger and smaller organisations are underrepresented in the study because they might not (yet) cooperate with such organisations. This might also account for diaspora organisations, which risk being underrepresented in the network of PDI support organisations. For Belgium and the Netherlands, we could compare the composition of our sample with those of earlier PDI studies conducted among a broader population of PDIs, and we did not find significant differences between our sample and those of these previous studies in terms of core characteristics of PDIs, such as average annual budget or number of members.

Because we did not include all existing PDI support organisations in our study, this might result in a bias with certain type of PDIs being underrepresented. In France there is a support organisation, FORIM (Forum des Organisations de Solidarité internationale issues de l’Immigration), that specifically reaches out to PDIs run by people from the diaspora community. Because no systematic comparison between those PDIs run by representatives of diaspora communities and those that are not, it is hard to see if and how this might have distorted the findings of our study. Because we included the largest support organisations in each country, we believe that we managed to reach out to a large number of representative PDIs in each country.

Although we conclude that PDIs so far are resilient and committed to continuing their work, a prolonged continuation of the crisis might raise concerns. With the personal encounter being at the heart of PDIs’ work, prolonged travel restrictions might prevent PDIs and their local counterparts from meeting in person. These personal encounters are known to have a motivating role for PDI members and a catalysing role for (mobilising) their (private) donors. Continued travel restriction might thus have a severe impact on the functioning of PDIs, also because it might affect the often close personal relationships, often referred to as friendships by those involved, between staff members of the PDI and its local partner. These friendships have been shown to impact the development of partners in the Global South (Kumi & Copestake, 2021). Additionally, in case the impact of the crisis continues to affect societies in the Global North, this might challenge the abilities of PDIs to raise funds from regular (private) donors to reach out to new (private) donors. In addition, a longer-lasting COVID-19 crisis might impact the visibility of PDIs in their own communities, not only affecting their fundraising activities and the development interventions they support, but also the role they play in contributing to global citizenship in their home countries.

The first policy implication of our findings relates to the opportunities the crisis provides for enhancing ownership of local partners. A longitudinal study on the development interventions of PDIs has shown that in the partnership between PDIs and their local partners overall little progress has taken place in terms of increased local ownership (Kinsbergen et al., 2020b). The results of our study show that during the COVID-19 crisis, local partners seized the opportunity to step up the game. Our study also shows that, for those PDIs and their local counterparts working on an exit strategy, the COVID-19 crisis may result in drawback in this process. With fewer opportunities for local fundraising, PDIs mentioned how they, after a process of reducing their financial and non-financial support, have to step forward again for their local counterpart to continue providing support. Knowing that many local partners are often heavily dependent on PDIs and that most PDIs do not have an explicit exit strategy or only started working on it at a very late stage (Kinsbergen et al., 2020a, 2020b, 2020c, 2020d), has meant that local counterparts are often ill prepared for an actual aid withdrawal. In times when the call for shifting power sounds louder than ever, it is time for PDIs, their local counterparts and support organisations to work on increased independence for the local counterparts, with the importance of the personal connectedness being a critical challenge in achieving this.

The second policy implication considers the importance of diversifying the fundraising strategies of organisations. Unlike some previous studies (Tuckman & Chang, 1991), but in line with Kinsbergen et al., (2020a, 2020b, 2020c, 2020d) and Chikoto and Neely (2014, p. 580), we found that PDIs benefit from ‘fundraising concentration’ with a larger number of revenue sources resulting in a more negative impact of the crisis. However, the results of our study showed that, to become more resilient, PDIs would gain by a strengthened online fundraising strategy. PDIs might be challenged in this, considering that older members may hamper PDIs’ ability and willingness to adapt offline fundraising strategies into a durable online strategy.

In terms of further research, the impact of current travel restrictions merits attention. It takes away an important motivating factor for PDI volunteers and, indirectly, their donors. The question hence is how all this impacts the local organisations and development interventions (co) funded by PDIs in the longer term. In addition, personal encounters between individuals from the Global North with individuals, communities or organisations in the Global South form the most important incentive for starting a PDI. Therefore, in the longer term, the question remains: how will this crisis impact the community of PDIs in broader terms, with currently no, or only a limited number of, new PDIs being established?

Let us return to the introduction, where we showed the substantial impact of the COVID-19 crisis on charitable organisations in general. While there are similarities in terms of the negative impact on organisations (i.e. fundraising challenges), there were also similarities with respect to resilience. However, there were also differences between the wider sector and PDIs, particularly those related to the important role of physical encounters between PDIs and their local partners. When analysing the impact of COVID-19 on charitable organisations, it remains important to engage in granular research and provide sufficient attention to the subsets of PDIs.

## Data Availability

Data collection took place in line with the GDPR.

## Appendix 2: Background information on organisations

**Table:**

Nr	NL/BE	Founding year	Intervention	Project-country	Core-members*	Annual budget
1	BE	2002	Mainly focusses on the improvement of (access to) education for children and young adults	Peru	2	€ 50,000
2	NL	2004	Several projects with a different nature to support the residents of the province of Nyanza	Kenya	3	€ 24,738
3	NL	2016	Socio-cultural projects for children	Brasil	5	€ 45,000
4	NL	1973	Supports the construction and maintenance of orphanages	Indonesia	5	€ 50,000
5	NL	1996	Focusses on the improvement of the health care and the training of medical staff	Uganda	5	€ 115,000
6	NL	2012	Several projects aimed at the development of children who live in townships	South-Africa	6	€ 30,000
7	BE	2019	Support of socio-cultural projects	Senegal	6	€ 3936
8	BE	2009	Improving education and healthcare for children with a disability	India	8	€ 15,000
9	NL	2007	Projects of a facility nature aimed at education and the development of children	India	10	€ 50,000
10	NL	2010	Projects of a facility nature aimed at education and healthcare	Ghana	12	€ 99,399
11	BE	2002	Several projects (orphanage, schools, pharmacy, bakery, agriculture) to support the citizens of a city in Congo, mostly aimed at children and healthcare	Democratic Republic of Congo	12	€ 31,412
12	NL	1991	Reducing poverty and improving education and healthcare	Peru	15	€ 170,000

*None of the organisations indicated to have paid-staff, thus all the active core-members are working as volunteers

## Footnotes

1 Staff members refer to both paid and non-paid staff. With the majority of PDIs being mostly run by volunteers only, in most cases this refers to volunteers.

2 Eu can aid! provides direct support to southern-based development organisations and does not provide support to northern-based PDIs and therefore did not participate in this study.
